# Cancer and the Quakers

**Published:** 1902-11-01

**Authors:** 


### CANCER AND THE QUAKERS.

It is being constantly borne in upon us how careful we ought to be in accepting statistical evidence as to medical affairs, especially when the collection of such statistics has extended over so long a time as to have a given opportunity for change of fashion in nomenclature or in classification to have occurred during the period in question. It is of the very essence of statistics that all the units in each category should be of strictly similar nature, a matter very difficult to ensure when the very definitions of diseases change as rapidly as they have done of late years. Hence we by no means feel inclined to accept at once the suggestions made by Mr. Jonathan Hutchinson in regard to the relation between arsenic and cancer. Still there is a good deal that is curious and interesting in what he says. He points out that there is a wide difference between the tale told by the Friends' Provident Institution and that laid bare by Scottish Widows' Fund and also by the general statistics of the country, for while in the Scottish Widows' Fund the same increase in the deaths registered as "cancer" has occurred which has been observed among in the community at large, in the Friends' Provident Institution no increase whatever has been observed. Now what is the difference between the Quakers and the rest of the world? Mr. Hutchinson suggests that the difference which is of importance in regard to the matter in hand may be expressed by the words "beer and tobacco." It has been proved, he says, that in men, amongst whom the great increase of cancer has taken place, it is the digestive tract which almost exclusively suffers. This tract suffers much less in women. "It is impossible then," he says,[1] "to avoid a suspicion that men in the United Kingdom, and even more so in New Zealand, have in recent times, with increasing frequency, been taking something into their mouths which is causative of cancer." What then is there that men take and women do not take, that men largely indulge in but Quakers do not? What but beer and tobacco? Now let us turn to history. Mr. Hutchinson says that the introduction of gas as an illuminant and the consequent supply of gasworks' coke as an article of commerce have been coincident with increased prevalence of cancerous disease. "It is from this source that almost all beer has come to contain arsenic in variable and usually quite insignificant quantities, but sometimes in such as are distinctly injurious. It is by no means improbable that the same thing has been going on in reference to kiln-dried tobacco." Meanwhile "it may be considered as proved" that chimney-sweepers' cancer is caused by the local irritation of arsenic contained in soot, and it "may be taken as well established" that the habit of smoking is productive of cancer, although we do not as yet know that this result is reached by way of arsenic. We confess that we should like to have more statistics and perhaps a little less hypothesis. Meanwhile we record these matters as indicating one at least of the lines of thought or perhaps of imagination which are at present drifting about in people's minds. Let us not be too hard upon imagination, which is the very salt of science.

[1] Polyclinic, Oct., 1902.

